# The Metric System in a Nut-shell

**Published:** 1878-08

**Authors:** Edward Wigglesworth


### Selected.

### THE METRIC SYSTEM IN A NUT-SHELL.

"Universality, Uniformity, Precision, Significance, Brevity and Completeness. A system of weights and measures born of philosophy rather than of chance."—*Charles Sumner.*

By Edward Wigglesworth, M. D.

"Washington, May 3.—Surgeon-General Woodworth, of the U. S. Marine Hosp. Service, has issued a circular, with the approval of Secretary Sherman, requiring medical officers of the Marine Hosp. Service to make use hereafter for all official, medical and pharmaceutical purposes, of the metric system of weights and measures, which had already, under the act of July 28, 1866, been adopted by this service for the purveying of medical supplies."—*Boston Daily Advertiser, May* 4, 1878.

The metric system is already *legalized* in both America and England. The only question now is, which of the two, the most progressive or the most conservative nation on earth, shall be the first to definitely and finally adopt it as an *exclusive* system? [N.B.—England was 400 years behind the continent in adopting our present arithmetic.] Russia has already taken the preliminary steps towards its final adoption. The rest of the civilized world long since made the system obligatory, in whole or in part, except that, in Sweden alone, its obligatory use is to date from a period in the future, 1889.

Now, what is this metric system? Metric, is from the Greek word "metron," a measure, spelled with Epsilon (e short) and, therefore, pronounced mĕt-ric:

The meter (measure) is, practically, a fixed quantity, namely, the ten millionth part of the earth's quadrant from the Equator to the North Pole. With the meter everything can be *measured,* for it is itself the unit of length; a cube, the edge of which is the tenth of a meter, is the unit of capacity (liter) and the weight of a cube of rain water, at its extreme contraction, the edge of which cube is a hundreth of a meter, is the unit of weight (gram).

It is the gram alone which concerns physicians, for, in the metric system, *everything is best prescribed and dispensed by weight alone;* numbers upon a prescription paper being regarded by the pharmacist as representing grams, unless the contrary is expressly stated. The fractions are always decimal.

The table is easily learned. It consists of six words. as prefixes, whether we deal with grams, liters, or meters. These are: Deci for tenth, centi for hundreth, milli for thousandth; deka for ten, hekto for hundred, kilo for thousand. Having these few words, the terms of Troy, Avoirdupois, and Apothecaries' weight, and of liquid measure, may be relegated to the limbo of pounds sterling, shillings, four-pence-ha'pennies, and farthings. As we say, dime, cent, mill, so we say decigram, centigram, milligram. These prefixes are Latin, and *diminish* the value. Deka, hekto, and kilo are Greek and *increase* the value. The mnemonic is G I L D, *i. e.*, Greek increases, Latin decreases. Deka occurs in the English word decade; hekto in hecatomb; kilo in chiliad.

"Being accustomed to the words mill, cent and dime, we shall find the words 'milligram,' 'centigram,' and 'decigram' quite as simple and easy to pronounce as our words 'pennyweight-troy,' 'hundrethweight-avoirdupois,' 'scruple-apothecaries,' etc., notwithstanding the assertion to the contrary of those who grieve to give up the 'short and sharp Anglo-Saxon words, used in our present *familiar* old tables of weights and measures."

Practically, moreover, for physicians, the whole system is reduced to grams and centigrams, just as, in money, to dollars and cents. On the right side of the prescription paper draw a perpendicular line from top to bottom. This decimal *line* takes the place of all the decimal *points*, and obviates the possibility of mistakes. This is the way dollars and cents are separated on business papers. Additional security is gained by writing the decimal fraction (centigrams) of half-size and raised above the line (of grams,) since it represents a numerator of which the denominator 100 is omitted. To make assurance doubly sure, "grams" may be written over the integer-column of figures, and, if wished, the word "decimals" over the decimal column.

Now, what is a gram, or rather, the values, metrically expressed, of our present awkward weights?

| | Prussian. | Practical. | Precise. |
|---|---|---|---|
| Grain I = | 0.06 | 0.06 | 0.065 |
| ℈ I = | 1.25 | 1.25 | 1.29 |
| ʒ I = | 3.75 | 4.0 | 3.89 |
| ℥ I = | 30.0 | 32.0 | 31.1 |

The "practical" table alone concerns us. The "Prussian" (by order of the Prussian Ministry, Aug. 29, 1867) is given merely to show that our table is even nearer the actual truth than one which has been proved by actual experience to answer every purpose. The values of the grain and scruple are a little too small. As they are used for powerful drugs this is an error in the right direction. The values of the drachm and ounce are a trifle too large, but the proportions and therefore the ratio of drug to vehicle are preserved.

A prescription written metrically is always proportionate, and whether the pharmacist uses pennyweights, pounds or tons; gills, pecks or chaldrons; pints, gallons or hogsheads, the ratios are preserved, and a teaspoon dose contains the same amount of medicine.

As regards administration, a teaspoon represents five grams, a tablespoon twenty grams; for a teaspoon holds one and one-third fluid drachms, a tablespoon a trifle more than four times as much.

In the metric system *everything is weighed*, thus obviating the difficulties of evaporation, refraction and adhesion, and obtaining more conveniently, more exact results. In our old "systemless system" some fluids were measured. How shall we obtain with weights, the desired bulks of fluids with varying weights? Must we learn the specific gravities of all fluids?

*Not at all!*

1. Fixed oils, honey, liquid acids and chloroform, must at present be prescribed in our old weights, not measures, according to the pharmacopœia. Here change old weights to metric ones.

2. Not enough chloroform or ether is included in any one prescription to admit of harm arising from the amount contained in a single dose, even were their weights regarded as the same with that of water. Moreover, it is not difficult to remember that ether weighs seven-tenths as much as water, chloroform twice as much as ether.

3. There remain infusions and tinctures, glycerines and syrups. These four are used in bulk as doses, or as solvents or vehicles. The former two may be regarded as identical in weight with water ; the latter two as one-third heavier, and when prescribing these we need merely write, by weight, for four-thirds as much as we should write for were we prescribing water, and we obtain an equal bulk. The teaspoon or tablespoon dose will then contain the desired amount of the drugs employed.

Or, simplest of all, we can make any mixture up to any desired bulk by merely directing the druggist to use enough of the vehicle to bring the whole mixture up to the requisite weight for that bulk.

The Metric Bureau, 32 Hawley street, Boston, will furnish metric prescription-blanks to order, to druggists or physicians at four-fifths printer's rates, or any blank can be made sufficiently metric by a perpendicular line at the right, headed *grams*.

---

Treatment of Strangulated Hernia by Ergotine.—Two cases are reported where ergotine was used as a dernier resort before operating. The skin over the tumor was, in each case, washed with an alkaline solution, to facilitate absorption, and pure ergotine was then rubbed in over it every two hours, while it was at the same time exhibited internally. After a few hours the distressing symptoms were relieved, and the hernia either subsided or was easily reduced.—*Le Bordeaux Médical.*

